# Participation in and use of skills development for work ability and expected retirement age: a cross-sectional study among senior workers

**DOI:** 10.3389/fpubh.2025.1511204

**Published:** 2025-02-18

**Authors:** Karina Glies Vincents Seeberg, Sebastian Venge Skovlund, Emil Sundstrup, Ole Steen Mortensen, Lars Louis Andersen

**Affiliations:** ^1^Musculoskeletal Disorders and Physical Work Load, The National Research Centre for the Working Environment, Copenhagen, Denmark; ^2^Department of Public Health, Section of Social Medicine, University of Copenhagen, Copenhagen, Denmark; ^3^Department of Sports Science and Clinical Biomechanics, University of Southern Denmark, Odense, Denmark; ^4^Department of Occupational and Social Medicine, Holbaek Hospital, Holbaek, Denmark; ^5^Department of Health Science and Technology, Aalborg University, Aalborg, Denmark

**Keywords:** adult education, senior workers, human capital theory, age dependency ratio, job supply, gerontology

## Abstract

**Introduction:**

Europe’s aging population calls for ways to prolong working life. Skills development initiatives could potentially improve work ability and extend working lives and may thus be key to address this challenge. However, the role of skills development in relation to work ability and retirement age is still not fully understood.

**Aim:**

This study aims to investigate the association of skills development with work ability and expected retirement age among senior workers.

**Methods:**

In 2022, all State employees in Denmark aged 55 years or above (*n* = 53,673) received a web-based questionnaire, of which 42% were included in the analyses (*n* = 22,544). The questionnaire included questions about participation in skills development initiatives over the past 2 years, lifestyle and work environment, including work ability and expected retirement age. We employed Generalized Linear Models (GLMs), weighted for Union, sex and age, with multivariate adjustment to examine associations of participation in (1) courses, (2) formal education, and (3) other forms of skills development on work ability and expected retirement age.

**Results:**

Formal education showed associations with expected retirement age with a between-group difference of 0.68 years Confidence Interval (CI) (0.54 to 0.82). Conversely, other forms of skills development (peer-to-peer training or self-study) were most positively associated with work ability in specific models with a between-group difference of 0.20 years CI (0.16 to 0.24).

**Conclusion:**

Our findings suggest that participation in skills development is positively associated with work ability and expected retirement age. These findings underscore the need for targeted skills development programs, which may enhance workforce sustainability and help workers prolong their working life.

## 1 Introduction

Europe faces important changes in demographics, characterized by a growing proportion of older adult citizens (1, 2). This shift is leading to an increased age dependency ratio, where fewer younger individuals are available to support and finance welfare systems (3). As more citizens approach retirement age, increasing focus on ensuring an adequate labor supply becomes crucial. This demographic shift, combined with the rapid evolution of work requirements, underscores the necessity of retaining older workers and expanding workforce participation. Several strategies have been proposed and implemented to deal with these potential challenges to societies (4), including raising the retirement age (5) and enhancing workforce productivity. A key area of focus is ensuring a high level of employment and productivity across all age groups to sustain economic stability and social welfare systems.

A potential solution to maintain workers in the workforce is skills development. Skills development initiatives can play a role in enhancing work ability, which in turn may influence retirement patterns. Moreover; such initiatives may result in better opportunities for work tasks that are more suited to the aging worker, allowing them to continue contributing effectively in roles that align with their evolving capabilities (6, 7). Studies suggest that by improving workers’ skills, particularly those of older workers, it is possible to prolong their working lives and delay retirement (8, 9).

Previous research has indicated a link between skills development initiatives and extended working lives (8–13). However, the existing literature pertaining to the relationship between different forms of skills development, work ability, and expected retirement age remains highly limited and not fully understood. It is unclear whether all forms of skills development are equally effective in achieving this outcome. Such knowledge is warranted by politicians and workplaces and could help them design effective initiatives potentially leading to more sustainable working lives.

This study aims to investigate the influence of skills development on work ability and expected retirement age among senior workers aged 55 and above. By examining various forms of skills development, including courses, formal education and other forms of skills development, we seek to provide new knowledge into their association with work ability and expected retirement age.

## 2 Methods

### 2.1 Study design

This is a cross-sectional study using self-reported questionnaire data on both the explanatory and outcome variables. We followed the recommendations from The STROBE reporting guideline for transparency (14).

### 2.2 Settings and population

This cross-sectional study was conducted among Danish State employees aged ≥55 years. Currently employed workers from five different Unions—this includes professionals, administrative staff, service workers, educators, and individuals in specialized public service roles. By incorporating diverse job categories, the study captures a broad sample, enabling a comprehensive understanding of work conditions and experiences across various professions and organizational levels. The participants received a questionnaire survey designed about the participants’ experiences with skills development initiatives as well as lifestyle, health, and work environment factors. Participation in the study was voluntary, and the sample is therefore non-random, as it depends on individuals’ willingness to respond to the questionnaire. As we did not have access to data on non-respondents, it was not possible to conduct a non-response analysis to determine whether the characteristics of the study sample differ significantly from those of the full sample.

### 2.3 Data collection

Data collection was conducted in 2022 using a questionnaire. The Danish Agency for the Modernization of Public Administration (MEDST) identified and drew all Civil Registration Numbers (CPR), which is a unique personal identification number assigned to all residents of Denmark, used for official identification and public services such as healthcare, taxation, and social security, for Danish State employees aged 55 years and above. These individuals were then contacted via a secure digital mail system, e-Boks used in Denmark to send official correspondence from both public institutions and private companies which is widely. The e-Boks system is linked to the CPR number assigned to each citizen, ensuring the secure and personalized distribution of the questionnaire.

**Figure 1:**
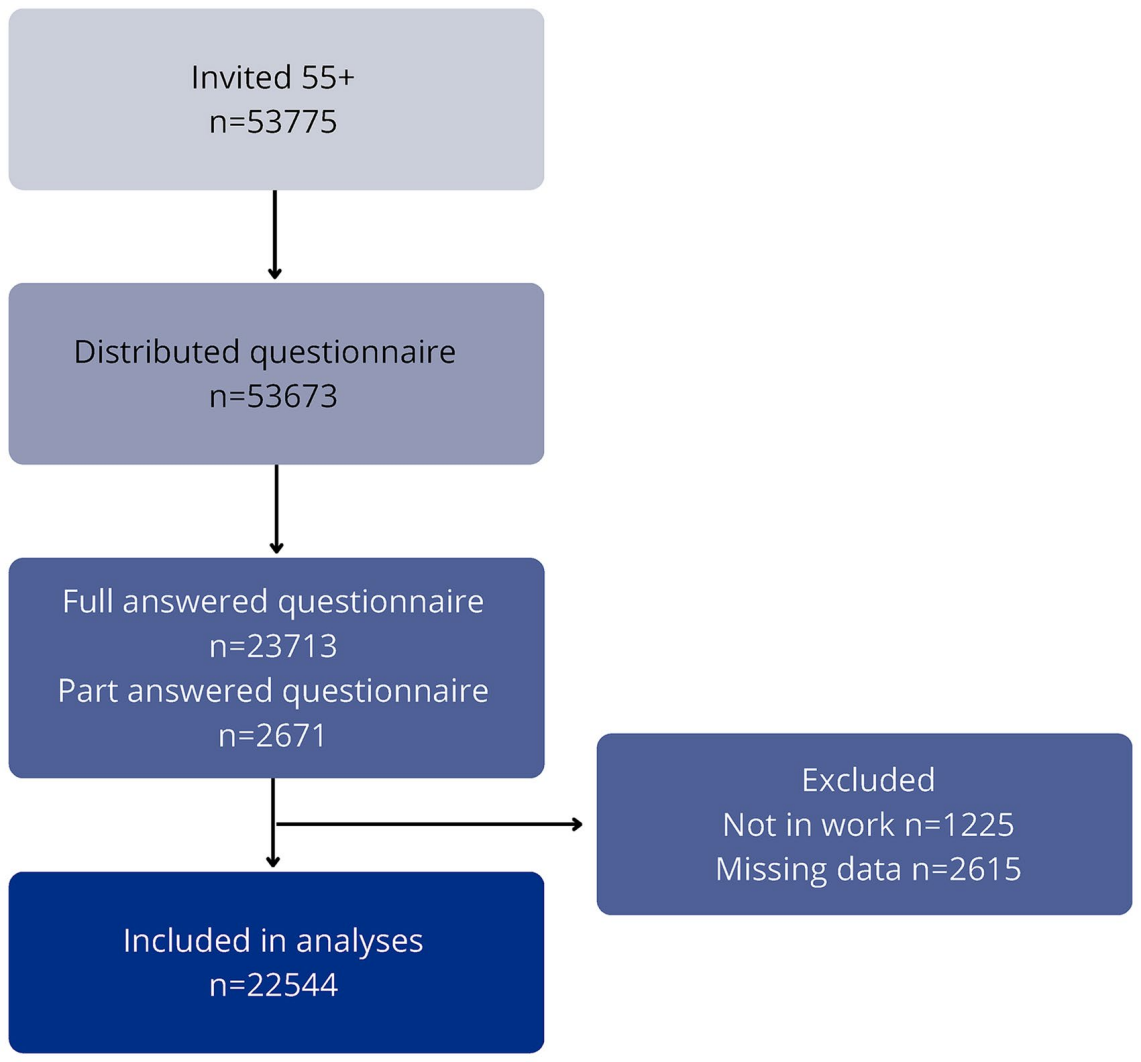
Flowchart participants.

The data collection spanned approximately 3 months. In case of non-response, reminders were sent to 2,929 individuals on October 25, 2022 to encourage participation. These reminders were disseminated through the same secure e-Boks system. The flow of participants throughout the data collection process is illustrated in Figure 1.

### 2.4 Measures

#### 2.4.1 Explanatory variables

##### 2.4.1.1 Skills development

The skills development measures were developed through a collaborative process involving The Secretariat for Competence Development (Kompetencesekretariatet) and representatives from both employer and employee organizations. The categories were specifically designed to reflect the complete range of skills development opportunities available within the Danish state sector, ensuring content validity for this specific context. The three distinct categories—courses, formal education, and other forms of skills development—were created to encompass both traditional and contemporary approaches to professional development, allowing for a comprehensive assessment of skills development participation among state employees.

Data on skills development were collected using the question; “Have you participated in or completed any of the following forms of skills development within the last 2 years?” The participants were provided with detailed explanations in each category of skill development, clarifying what was meant by courses, formal education, and other forms of skill development, from which they could choose. This includes both in-person and online activities, and participants could select multiple categories of skills development:

###### 2.4.1.1.1 Courses

This includes short or long-duration courses, whether conducted online or in-person.

###### 2.4.1.1.2 Formal education

Encompasses formal educational programs ranging from individual modules to full degrees (e.g., bachelor’s, master’s, Ph.D., vocational training).

###### 2.4.1.1.3 Other forms of skills development

This includes informal learning activities such as mentoring, peer learning, conferences, seminars, self-study, and other non-traditional educational experiences.

#### 2.4.2 Outcomes

##### 2.4.2.1 Expected retirement age

The expected retirement age was assessed using the following question: “At what age do you expect to permanently leave the labor market?” This single-item measure provided a direct estimate of each participant’s anticipated age of retirement. We have utilized this question in previous research (15, 16).

##### 2.4.2.2 Work ability

Work ability was assessed using a single-item question from the Work Ability Index (WAI): “Please rate your current work ability on a scale of 0–10, where 0 is unable to work and 10 is lifetime best work ability.” The Work Ability Index (WAI) scale ranged from 0 to 10 and was treated as a discrete variable. For descriptive purposes in Table 1, we categorized the WAI scores into three groups: poor (0–5), moderate (6–7), and good/excellent (8–10), to provide an overview of the distribution among participants (17, 18).

**Table 1:** Characteristics of the included sample.^1^

Variable	*n*	Percent (%)
Age		
55–59	11,129	49.4
60–64	8,455	37.5
65+	2,960	13.1
Sex		
Male	11,543	51.2
Female	11,001	48.8
Workplace location (demographics)		
Capital Region	8,409	37.39
Central Denmark Region	4,757	21.15
North Denmark Region	2,178	9.68
Region Zealand	2,911	12.94
Region of Southern Denmark	4,237	18.84
Smoking		
Yes	2090	9.3
No	20,326	90.7
BMI		
Underweight (< 18.5)	217	1.0
Normal weight (18.5–24.9)	8,873	41.2
Overweight (25–29.9)	8,745	40.6
Obesity (30 and above)	3,682	17.1
Physical activity in leisure time		
Reads, watches television, or engages in other sedentary activities	1863	8.3
Takes walks, cycles, or engages in other light physical activities for at least 4 h per week	14,440	64.4
Participates in structured exercise or performs heavy gardening or similar activities for at least 4 h per week	5,636	25.1
Engages in intense training and regularly participates in competitive sports several times a week.	501	2.2
Physical activity at work		
Most sedentary work, which does not require physical exertion	14,079	62.5
Mostly standing or walking work, which otherwise does not require physical exertion	6,336	28.1
Standing or walking work involving some lifting or carrying.	1832	8.1
Heavy or fast-paced work that is physically demanding.	297	1.3
Unions^2^		
AC	7,697	34.1
CO10	4,377	19.4
LC	2,968	13.2
OAO	7,021	31.1
Others	481	2.1
Health		
Good to Excellent	20,077	89.3
Poor to Fair	2,409	10.7
Do you know exactly what your work tasks are?		
1. Always or often	20,166	89.45
2. Sometimes	1877	8.33
3. Never or rarely	501	2.22
Do you and your colleagues recognize each other at work?		
1. Always or often	16,705	74.1
2. Sometimes	4,402	19.53
3. Never or rarely	1,437	6.37
Do you have influence on how you perform your work tasks?		
1. Always or often	19,607	86.97
2. Sometimes	2,213	9.82
3. Never or rarely	724	3.21
Do you have influence on when you perform your work tasks?		
1. Always or often	15,833	70.23
2. Sometimes	4,394	19.49
3. Never or rarely	2,317	10.28
Does your job give you confidence and job satisfaction?		
1. To a high degree	14,606	64.79
2. To some degree	6,171	27.37
3. To a lesser degree	1767	7.84
Participated in or completed any of the following forms of skills development		
Courses	10,522	46.7
Formal education	1803	8.0
Other forms of skills development	10,532	46.7
Not participated in any form of skills development	6,470	28.7
At what age do you expect to leave the workforce completely?		
65 years or younger	7,363	33.4
66–67 years	6,168	28.0
68–69 years	4,050	18.4
70 years or older	4,482	20.3
Work ability		
Good work ability (8–10)	16,330	72.4
Moderate work ability (6–7)	4,728	21.0
Poor work ability (0–5)	1,486	6.6

1 The total number of participants included in the sample is 22,544, which represents 42% of the initial sample. The remaining participants did not respond.

2 AC: Academic Central Organization—Represents professionals with higher academic qualifications in various sectors. OAO: Public Employees’ Organization—Represents unions for public sector employees. LC: Teachers’ Central Organization—Represents teachers and educational staff in Denmark. CO10: Central Organization 2010—Represents civil servants in various administrative roles within the public sector.

Although the full WAI index includes multiple items, previous studies have also used this single item to assess work ability effectively (19). Using a single-item approach simplifies the data collection process, which can be advantageous in large-scale studies by reducing respondent burden and potentially increasing response rates.

The Work Ability Index (WAI) was chosen for its ability to measure work ability comprehensively by considering various dimensions, including physical health, mental well-being, and alignment between work demands and individual capacity (20).

#### 2.4.3 Potential confounding factors

##### 2.4.3.1 Age and sex

Age and sex were determined using participants’ CPR numbers. Evidence indicates that work ability tends to decline with age, with older workers potentially adjusting their expected retirement age due to declining health or reduced job satisfaction (21, 22). Age also influences opportunities for skill development, with younger workers often having greater access compared to their older counterparts. Similarly, sex plays a significant role in work ability and retirement expectations. Women, for instance, may retire earlier due to caregiving responsibilities, lower lifetime earnings, and sex-based wage gaps (23). These factors can limit professional growth opportunities for women, further influencing retirement expectations (23).

##### 2.4.3.2 Health/general health

Health data were collected by asking participants about their height, weight and smoking habits smoking (yes/no). Physical activity during leisure time was assessed with the question; “In the past year, which of the following best describes your physical activity during your leisure time?” The response options were: (1) Reading, watching television, or engages in other sedentary activities, (2) Walking, cycling, or performing other light physical activities for at least 4 h per week, (3) Participates in structured exercise or performs heavy gardening or similar activities for at least 4 h per week, or (4) Participating in intense training or competitive sports several times a week. Self-rated health was determined by using a 5-point scale; poor, fair, good to excellent from the COPSOQ II questionnaire (24). Poor health is linked to earlier retirement, as chronic conditions or functional limitations make work more difficult (25). Those in better health are more likely to engage in skill development, which can enhance employability and extend working life (26).

##### 2.4.3.3 Physical demands at work

Physical demands at work were assessed using the question: “how will you describe your general physical activity at work? Mostly sedentary work that does not require physical exertion, mostly standing or walking work that otherwise does not require physical exertion, standing or walking work with a fair amount of lifting and carrying, or heavy or fast-paced work that requires physical exertion. Physically demanding jobs were considered a potential confounder, as they are linked to reduced work ability, especially in older workers (27). These jobs can lead to earlier retirement due to physical strain (25, 28) and offer fewer opportunities for skill development, which can reduce the ability to prolong working life.

##### 2.4.3.4 Psychosocial work factors including role clarity, influence at work, recognition and job satisfaction

Participants provided information on psychosocial work factors such as role clarity, influence at work, recognition, and job satisfaction, in part through the COPSOQ II questionnaire (24). To assess role clarity, we asked participants the following question: “Do you have a clear understanding of your work tasks?” with response options ranging from “always,” “often,” “sometimes,” “rarely,” to “never.” Recognition was measured by asking: “Do you and your colleagues acknowledge each other’s work?” using the same response scale. Influence at work was assessed with the question: “Do you have influence on how you perform your tasks?” with possible responses ranging from “always” to “never.” We also asked: “Do you feel that your work is recognized and appreciated by management?” with similar response options. Finally, job satisfaction was evaluated by asking: “Does your work give you confidence and job satisfaction?” with responses ranging from “to a very high degree,” “to a high degree,” “to some degree,” “to a lesser degree,” to “not at all.

Poor psychosocial working conditions, such as low recognition and job satisfaction, can push individuals toward early retirement by reducing work ability and increasing job strain (29). In contrast, a positive work environment can support skill development and prolong working life. Research shows that improving these factors can enhance work ability and delay retirement (30, 31).

### 2.5 Statistical analyses

SAS version 9.4 (SAS Institute, Cary, North Carolina, USA) were used for statistical analysis. We used the GLM (General Linear Model) procedure to calculate least squares mean differences between skills development vs. no skill development and work ability or expected retirement age. Model-assisted weights were used for the SurveyFreq and SurveyLogistic procedures to produce representative estimates and 95% confidence intervals. These weights were based on age, sex, unions.

“We assessed the normal distribution of our continuous variables and verified the assumptions of the Generalized Linear Model (GLM) by evaluating residuals using histograms, residual plots, and Q-Q plots.”

We controlled for age, sex, BMI, physical demands at work, smoking, physical activity in leisure time, general health and psychosocial work factors including role clarity, influence at work, recognition and job satisfaction, in our main analysis. These confounders were selected based on Spearman correlation and previously demonstrated associations between these factors and skill development (23, 30, 31), work ability (21, 27, 29–31), and/or expected retirement age (22, 26, 28–31) are discussed in the section above.

We conducted a stratified analysis to examine subgroup differences. The analysis was stratified by age and sex, guided by existing literature, and adjusted for organizational affiliation, smoking, leisure-time physical activity, and BMI. Age was divided into two groups: 55–59 years and 60 years and older, reflecting key differences in labor market participation and life stages. The 55–59 age group typically includes individuals who are still active in the workforce but may start considering retirement. In contrast, the 60+ group represents a transition phase where many either retire or significantly reduce their workforce participation (23, 32). Within age groups we additionally, controlled for age as a continuous variable.

#### 2.5.1 Sensitivity analyses

We conduct sensitivity analyses based on existing literature to assess the robustness of our findings regarding sedentary work as a predictor of educational attainment and our outcomes. Specifically, these sensitivity analyses explore whether variations in the sedentary work variable influence the relationships observed with educational level and self-reported health.

## 3 Results

We analyzed data from 22,544 participants. Table 1 presents the characteristics of the included participants. It shows a nearly equal representation of male and female participants (51.2 and 48.8%, respectively). The majority (49. 4%) of participants were within the 55–59 age group. Additionally, 89.3% perceived their health as good or excellent. Our findings indicated that our included sample predominantly has sedentary jobs as less than 10% stated that they had either Standing or walking work involving some lifting or carrying or heavy or fast-paced work that is physically demanding.

A large proportion of participants had engaged in some form of skills development, with only 28.7% reporting no participation. Courses and other skills development activities were particularly favored or offered to the participants. In total, 33.4% of workers expected to retire before the official retirement (67 years) age, while 38.7% of workers expected to continue working beyond the statutory retirement age. Moreover, 72.4% perceived their work ability as good or moderate, while 6.6% perceived their work ability as poor.

### 3.1 Skills development and work ability

Table 2 presents the results of the GLM assessing the association of skills development with work ability. The fully adjusted model shows a statistically significant association between all three forms of skills development and a higher level of work ability: courses 0.12 (95% CI 0.08 to 0.16), formal education 0.18 CI (0.11 to 0.26) and other forms of skills development 0.14 CI (0.10 to 0.18).

**Table 2:** Main GLM analysis of courses, formal education and other forms of skills development on work ability and expected retirement age cumulative modeling adjustment.

Outcomes	Exposure	Model 1^A^			Model 2^B^			Model 3^C^		
		Between-group mean diff. (95% CI)		*p* value	Between-group mean diff. (95% CI)		*p* value	Between-group mean diff. (95% CI)		*p* value
Work ability (0–10)	Courses	0.17	(0.13 to 0.21)	<0.0001	0.15	(0.11 to 0.19)	<0.0001	0.12	(0.08 to 0.16)	<0.0001
	Formal education	0.20	(0.13 to 0.28)	<0.0001	0.19	(0.12 to 0.26)	<0.0001	0.18	(0.11 to 0.26)	<0.0001
	Other forms of skills development	0.25	(0.21 to 0.29)	<0.0001	0.20	(0.16 to 0.24)	<0.0001	0.14	(0.10 to 0.18)	<0.0001
Expected retirement age (unit: years)*	Courses	0.12	(0.04 to 0.19)	0.002	0.11	(0.03 to 0.18)	0.006	0.09	(0.01 to 0.17)	0.020
	Formal education	0.69	(0.55 to 0.83)	<0.0001	0.68	(0.54 to 0.82)	<0.0001	0.68	(0.54 to 0.82)	<0.0001
	Other forms of skills development	0.26	(0.18 to 0.33)	<0.0001	0.26	(0.18 to 0.34)	<0.0001	0.22	(0.14 to 0.30)	<0.0001

A Adjusted for sex, age and organizations.

B Further adjusted for smoking, physical activity in leisure time, physical demands at work, and BMI.

C Further adjusted for demographics, psychosocial work factors and health*..

#### 3.1.1 Skills development and work ability stratified analysis on age and sex

We performed stratified analyses to investigate the relationship between age, sex, skills development and work ability. Due to the risk of over-adjustment in Model 3, where psychosocial work factors could act as either a mediator or a collider, we chose to use Model 2. Psychosocial factors might mediate the associations of skills development on work ability by improving aspects like job satisfaction and stress levels, which in turn enhance work ability. Alternatively, if these factors influence both skills development and work ability, adjusting for them could distort the true relationship. Thus, we opted for Model 2 to provide a more accurate assessment of the link between skills development and work ability. Skills development remained associated with work ability for all stratified groups, except for older men and formal education. Significant associations were found for formal education among younger men 0.26 (CI 0.13 to 0.40) and older women 0.28 (CI 0.09 to 0.47) and other forms of skills development among younger women 0.24 (CI 0.15 to 0.32) and older men 0.18 (CI 0.11 to 0.25) (see Table 3).

**Table 3:** Stratified analysis of skills development stratified on sex and age (blow and above 60 years old) on work ability and expected retirement age.

Outcomes	Exposures	Model 2 stratified^A^											
		Younger men			Younger women			Older men			Older women		
		Between-group mean diff. (95% CI)		*p* value	Between-group mean diff. (95% CI)		*p* value	Between-group mean diff. (95% CI)		*p* value	Between-group mean diff. (95% CI)		*p* value
Work ability (0–10)	Courses	0.19	(0.11 to 0.27)	<0.0001	0.15	(0.07 to 0.24)	0.0003	0.16	(0.09 to 0.23)	<0.0001	0.11	(0.02 to 0.19)	0.0129
	Formal education	0.26	(0.13 to 0.40)	0.0001	0.16	(0.03 to 0.29)	0.0148	0.05	(−0.12 to 0.21)	0.574	0.28	(0.09 to 0.47)	0.0033
	Other forms of skills development	0.19	(0.10 to 0.27)	<0.0001	0.24	(0.15 to 0.32)	<0.0001	0.18	(0.11 to 0.25)	<0.0001	0.18	(0.09 to0.27)	<0.0001
Expected retirement age (Unit: years)	Courses	0.14	(−0.08 to 0.35)	0.205	0.13	(−0.04 to 0.31)	0.127	-0.11	(−0.29 to 0.08)	0.255	0.15	(−0.02 to 0.31)	0.077
	Formal education	0.57	(0.21 to 0.92)	0.002	0.80	(0.53 to 1.07)	<0.0001	0.67	(0.25 to 1.10)	0.002	0.85	(0.49 to 1.20)	<0.0001
	Other forms of skills development	0.43	(0.20 to 0.65)	0.0002	0.11	(−0.06 to 0.29)	0.206	0.23	(0.04 to 0.42)	0.016	0.02	(−0.15 to 0.18)	0.831

A Adjusted for organizations, smoking, physical activity in leisure time, physical demands at work, and BMI.

#### 3.1.2 Skills development and work ability sensitivity analysis on sedentary workers and good health

We conducted a sensitivity analysis focusing on sedentary workers with good or excellent self-reported health. Statistically significant associations were observed for skill development and work ability, though the mean differences were minor (see Table 4).

**Table 4:** Sensitivity analysis on skills development and sedentary workers with good self-rated health on work ability and expected retirement age.

Outcomes	Exposures	Model 2^A^			Model 3^B^		
		Between-group mean diff. (95% CI)		*p* value	Between-group mean diff. (95% CI)		*p* value
Work ability (0–10)	Courses	0.07	(0.02 to 0.11)	0.002	0.05	(0.01 to 0.09)	0.020
	Formal education	0.10	(0.02 to 0.18)	0.013	0.09	(0.01 to 0.17)	0.024
	Other forms of skills development	0.11	(0.07 to 0.16)	<0.0001	0.06	(0.02 to 0.10)	0.008
Expected retirement age (Unit: years)*	Courses	0.05	(−0.05 to 0.16)	0.297	0.06	(−0.04 to 0.16)	0.249
	Formal education	0.65	(0.46 to 0.84)	<0.0001	0.67	(0.48 to 0.86)	<0.0001
	Other forms of skills development	0.16	(0.06 to 0.26)	0.003	0.12	(0.01 to 0.22)	0.026

A Adjusted for age, sex, organizations, smoking, physical activity in leisure time and BMI.

B Adjusted for age, sex, organizations, smoking, physical activity in leisure time, BMI, demography, psychosocial work factors and health*..

### 3.2 Skills development and expected retirement age

In addition to exploring the association between skills development and work ability, we investigated the relationship between skills development and expected retirement age. Due to the varied plots observed regarding normal distribution, we opted to trim our data to focus solely on the expected retirement age between 55 and 80 years. This decision was informed by the plots demonstrating normal distribution within this age group.

We observed a significant positive association between skills development and expected retirement age across all models. The fully adjusted model shows a statistical significant association between course 0.09 years CI (0.01 to 0.17), formal education 0.68 years CI (0.54 to 0.82) and other forms of skills development 0.22 years CI (0.14 to 0.30) and higher expected retirement age (see Table 2).

#### 3.2.1 Skills development and expected retirement age: stratified analysis on age and sex

We performed a stratified analysis to investigate the relationship between age and sex for skills development, and expected retirement age. The analysis was based on Model 2, as Model 3 was deemed at high risk of being over-adjusted as mentioned in the previous section regarding stratified analysis. For all stratified groups; younger men 0.14 years (−0.08 to 0.35), younger women 0.13 years (−0.04 to 0.31), older men−0.11 years (−0.29 to 0.08) and older women 0.15 years (−0.02 to 0.31), courses were not significant. For both younger (59 years old and below) 0.11 years (−0.06 to 0.29) and older women (60 years old and above) 0.02 years (−0.15 to 0.18), other forms of skills development were not significant as well. Formal education and expected retirement age were significantly positively associated, with a mean difference of younger men 0.57 years (CI 0.21 to 0.92), younger women 0.80 (CI 0.53 to 1.07), older men 0.67 years (CI 0.25 to 1.10) and older women 0.85 years (CI 0.49 to 1.20) (see Table 3).

#### 3.2.2 Skills development and expected retirement age: sensitivity analysis

Whe sensitivity analysis were focusing on sedentary workers with good self-reported health (good and excellent). Courses were not significant in ether models, while other forms of skills development revealed statistically significant. Formal education remained statistically significant, indicating robust findings [Model 2: 0.65 (CI 0.46 to 0.84), Model 3: 0.67 (CI 0.48 to 0.86)].

## 4 Discussion

### 4.1 Main results

Our study found that skills development is positively associated with both work ability and expected retirement age among state employees aged 55 and above. Notably, formal education was significantly associated with expected retirement age, see Table 2, while other forms of skills development were significantly linked to work ability see Table 2, model 1 and 2. These findings underscore the importance of targeted skills development programs as a means to potentially enhance work ability and extend working lives for senior workers. By focusing on improving specific types of skills, employers and policymakers could support older workers in maintaining their capacity to work and adjust their retirement expectations.

Although our study is cross-sectional, meaning causality cannot be established; our results are consistent with those of other cross-sectional and prospective studies, which have demonstrated positive relationships between skills development and work ability and expected retirement age. Previous cross-sectional research has shown that skills development is associated with higher work ability (33) and prospective studies further suggest that engaging in skills development may contribute to reduce early or delay retirement (8, 9, 13, 34).

### 4.2 Statistical significance vs. practical relevance

While many of our results were statistically significant, it is important to assess their practical relevance. For instance, the association of courses on expected retirement age was relatively modest compared to formal education. This underscores the need to prioritize the most beneficial forms of skills development in policies and practices. The association of formal education on expected retirement age indicate that investing in higher-level training could be particularly beneficial for prolonging working lives.

Our results show that the difference in work ability remained within less than one point. To evaluate the practical relevance of between differences in work ability, it is essential to establish a threshold that signifies a meaningful difference. Minor changes, such as 0.1 points, are insufficient to meet practical relevance criteria. Evidence suggests that a minimum change of one point in the Work Ability scale is required to achieve practical relevant outcomes (19, 21, 35). A one-point reduction in the Work Ability scale increases the risk of long-term sickness absence by 15.1% and early retirement by 33%, while a one-point increase reduces the risk of mobility limitations among retirees (19, 21, 35). Consequently, for changes in work ability to be meaningful and clinically relevant, they must exceed one point. Even though we discuss the potential lack of clinical relevance in work ability, even small differences in expected retirement age could be significant when viewed from a broader social and economic perspective.

### 4.3 Job supply

Our main analysis revealed that receiving formal skills development is associated with a higher expected retirement age between 0.68–0.69 full-time equivalent (FTE) years depending on models. For those who have not received such training, this potential extension in work life could be significant. To illustrate the potential implications, if formal skills development were implemented across the sample, we could potentially increase the labor supply by a substantial number of FTE years. Specifically, this could result in an aggregate gain of (6.470*0.68) = 4,400 − (6,470*0.69) = 4,464 FTE years.

Considering other forms of skills development, we observed a higher expected retirement age in the main analysis between 0.22–0.26 years. This potentially translates to an increase in labor supply by 1,423–1,682 FTE years for those who have not received any form of skills development.

Our findings highlight the importance of implementing comprehensive skills development programs to extend working lives and enhance labor supply. This perspective shifts the focus from individual benefits to societal gains, demonstrating that offering skills development opportunities to those who currently lack them could result in many additional years of work and productivity.

### 4.4 Theoretical perspective

Our research is informed by several theoretical frameworks, including Human Capital Theory and Social Exchange Theory in the form of Reciprocity Theory. Together these frameworks provide a comprehensive understanding of the interplay between motivations, work ability, expected retirement age, and skills development in older workers.

Human Capital Theory emphasizes the importance of investing in skills and knowledge to enhance productivity and job security (36). This concept is reflected in our findings, where increased skills development is linked to higher work ability. Public policies inspired by Human Capital Theory often focus on promoting workplace training, lifelong learning subsidies, and incentives for employers to support skill development among workers. These policies are particularly critical for older workers, as they help maintain employability, improve productivity, and potentially delay retirement (37, 38). Access to training improves not only professional skills but also overall well-being (37, 39). By equipping older workers with the skills needed to adapt to technological changes and evolving job demands, these initiatives may also contribute to sustainable labor market participation and reduce pressure on public pension systems.

Additionally, Reciprocity Theory, rooted in Social Exchange Theory, highlights the reciprocal relationship between workers and employers. When workers perceive that their employers invest in their development, they often feel an obligation to reciprocate through increased loyalty and extended tenure (40–42). Our data align with this, showing a positive association between skills development and a higher expected retirement age, suggesting that workers who perceive employer investment in their development are more likely to extend their tenure. Studies like Venneberg and Wilkinson (43) and Li et al. (11) further emphasize the importance of utilizing older workers’ accumulated knowledge and fostering supportive organizational climates for skills development and retention. Integrating these perspectives underscores the mutual benefits of investing in older workers’ skills: while workers improve their productivity and career stability, employers benefit from increased retention and engagement.

### 4.5 Practical recommendations

For a practical approach to addressing the challenges of an aging workforce, it is essential to consider the implementation of effective skills development programs. To improve work ability and potentially extend the expected retirement age, skills development programs for senior workers could include several suggested features. These might involve customized training content addressing both job-specific and transferable skills, flexible delivery methods to suit individual needs, and supportive organizational structures to facilitate the application of new skills. Additionally, integrating health-promoting elements and offering incentives for participation may enhance engagement and effectiveness. For instance, workplace-based digital literacy training combined with peer mentoring could improve skills while fostering intergenerational collaboration and engagement. Based on our findings, we propose that employers and policymakers consider skills development, particularly formal education, as a potential strategy to extend working lives and improve work ability among older workers. However, it is important to recognize that our study only shows an association, not causation. The varying associations of different types of skills development suggest that a tailored approach may be beneficial. Workplaces could consider offering a mix of formal education and other skills development opportunities to maximize benefits for both work ability and extended working lives. Further research is needed to determine long-term outcomes and optimal implementation strategies, but our results provide a foundation for promoting skills development as a component of age management policies.

### 4.6 Strength and limitations

Our cross-sectional design provides valuable insights but also has limitations. The use of self-reported data may lead to recall bias in relation to participation in skills development and expectation bias in relation to expected retirement age. Additionally, the questions used to measure skills development were not validated, which may affect the results. Although the categories were developed in collaboration with relevant stakeholders, the lack of validation means we cannot fully confirm that the measures accurately capture the intended constructs of skills development in this specific context.

In terms of selection bias, while we were unable to perform a non-response analysis, our response rate of 42% is relatively reasonable, although it is somewhat lower than similar surveys of workers, such as the Work Environment and Health Study (AH study) (response rate of 60%) (44) and the Senior Working Life study (response rate of 62%) (16).We also applied weighting based on age, sex and organizations to account for non-respondents. Despite this, we cannot be certain of the characteristics of those who did not respond. Additionally, as participation in the study was voluntary, the sample is non-random and reflects only those who chose to participate. If, as studies suggest, non-respondents are primarily individuals with lower educational attainment, poorer work ability, and an earlier expected retirement age—those who may benefit most from skills development—this could further compromise the validity of our findings and restrict their generalizability to the broader population of Danish State employees aged 55 years and above.”

In our analysis, we considered the potential for over-adjustment in Model 3, particularly regarding psychosocial work factors and health. These variables could act as either colliders or mediators in the relationship between skills development and work ability or expected retirement age. If they are mediators, adjusting for them might obscure the true association of skills development. Conversely, if they are colliders, controlling for them could introduce bias and distort the observed relationship. Despite this, the results remain significant even when these variables are not adjusted for, suggesting that the potential influence of these factors as colliders or mediators may be less pronounced. Therefore, while Models 2 and 3 provide valuable insights, Model 2 may represent the most reliable approach given the potential complexities of over-adjustment in Model 3.

While we were unable to control for all relevant variables, such as educational level and socioeconomic status, we did account for union organizations, as described in the methods section. The anticipated variation in education levels among different employee groups—specifically AC (Academic Central Organization), CO10 (Central Organization 2010), LC (Teachers’ Central Organization), and OAO (Public Employees’ Organization)—reflects the unique requirements of each category. Typically, AC members hold advanced degrees, whereas CO10 and LC employees display a broader range of educational backgrounds, from secondary education to higher qualifications. OAO includes a diverse array of educational attainment levels, underscoring the varied nature of jobs and industries represented. This educational diversity can influence job responsibilities, career advancement opportunities, and overall job satisfaction across these groups. For instance, the significant association between formal education and expected retirement age may partially stem from existing differences in educational background and career opportunities.

Future studies with longitudinal designs and register-based measures could further strengthen our findings and help interpret these associations. While our study provides insights into workers’ intentions regarding retirement, we acknowledge that expected retirement age does not necessarily reflect actual retirement behavior. To address this, future research could examine the prospective association between skills development and actual retirement age, using longitudinal data and register linkage to clarify whether the positive associations observed in our study translate into actual changes in retirement patterns. Longitudinal studies are essential for exploring how changes in skills and work ability influence retirement timing. Additionally, register-based measures and intervention-based studies would offer valuable insights into the impact of targeted skills development programs on retirement patterns. Replicating these findings across different occupational and national contexts would also enhance our understanding of the generalizability of these associations.

A key strength of our study was that we conducted sensitivity and stratified analyses to assess the consistency of our results. These analyses allowed us to test the stability of our findings under various conditions and explore differences within subgroups. While caution is needed regarding the error estimates due to the use of split subsamples, the significant associations observed remained consistent, which supports the reliability of our conclusions.

## 5 Conclusion

Our study demonstrates a positive association between skills development and both work ability and expected retirement age among our sample of Danish State employees aged 55 years and above. Specifically, formal education exhibits the significant association with extending the expected retirement age; while other forms of skills development most significantly showed a positive association with work ability. However, as participation in the study was voluntary and the sample is therefore non-random, the findings should be interpreted with caution and cannot be directly generalized to all Danish State employees aged 55 years or above. Further replication studies are needed to assess the stability and broader applicability of these results. Despite this limitation, these findings highlight the potential of targeted skills development initiatives to enhance work ability and prolong working lives among older workers.

## Data Availability

The raw data supporting the conclusions of this article will be made available by the authors, without undue reservation.
